# Transferability of radiomics models between deep learning and conventional CT reconstruction algorithms: A task‐based assessment for stratifying acute pancreatitis severity

**DOI:** 10.1002/acm2.70551

**Published:** 2026-03-25

**Authors:** Xiaobao Hu, Xiao Liang, Zhongren Huang, Yanyan Zhu, Mengya Guo, Zhihua Wu, Fuqing Zhou

**Affiliations:** ^1^ Jiangxi Provincial Key Laboratory for Precision Pathology and Intelligent Diagnosis Department of Radiology the First Affiliated Hospital Jiangxi Medical College Nanchang University Nanchang China; ^2^ Jiangxi Province Medical Imaging Research Institute Nanchang China; ^3^ Clinical Research Center for Medical Imaging in Jiangxi Province Nanchang China; ^4^ CT Imaging Research Center GE HealthCare China Beijing China

**Keywords:** acute pancreatitis, computed tomography, deep learning image reconstruction, machine learning, radiomic robustness

## Abstract

**Background:**

Extensive evidence has demonstrated the superior image quality achieved through deep learning‐based reconstruction algorithms. However, given their growing adoption in clinical imaging, the impact of these algorithms on radiomics model development warrants thorough investigation.

**Purpose:**

To investigate the transferability of radiomics models for acute pancreatitis (AP) severity stratification across CT images reconstructed with filtered back projection (FBP), adaptive statistical iterative reconstruction‐Veo (ASIR‐V), and deep learning image reconstruction (DLIR) algorithms.

**Methods:**

This retrospective study enrolled 79 AP patients who underwent contrast‐enhanced CT. Five sets of images were reconstructed with FBP, ASIR‐V at 50% blending level (AR50), and DLIR at low (DLRL), medium (DLRM), and high (DLRH) strength levels. A total of 837 radiomic features were extracted from the pancreatic parenchyma volume to generate five corresponding datasets. Radiomics models for stratifying AP severity were built on four machine learning algorithms, trained on three DLIR datasets (source datasets) and tested on AR50 and FBP datasets (target datasets). The source and target datasets were then swapped for reverse evaluation. Model transferability across datasets was assessed by comparing performance differences between source and target datasets, with area under the receiver operating characteristic curve (AUC) as the primary metric.

**Results:**

We identified a fundamental asymmetry in model transferability. Models trained on conventional reconstructions (AR50, FBP) demonstrated robust transfer performance to all DLIR datasets, with AUC values in target datasets generally exceeding those in source datasets. In contrast, only models trained on the DLRL dataset exhibited non‐inferior transferability when transferred to both AR50 and FBP datasets, with target AUCs being comparable to or higher than those in source datasets.

**Conclusions:**

The transferability of radiomics models across CT reconstruction algorithms is asymmetric. Radiomics models demonstrated robust transferability from AR50 and FBP datasets to DLIR datasets for AP severity stratification.

## 1 INTRODUCTION

Radiomics, as a quantitative approach that extracts high‐dimensional, mineable data from medical images, has shown great promise in constructing diagnostic, prognostic, and predictive models to support clinical decision‐making.^1^ While oncological applications have dominated the field, its expansion to non‐neoplastic conditions such as acute pancreatitis (AP) provides an appropriate clinical context for investigating how technical factors influence model performance.^2^, ^3^ Despite this potential, the widespread clinical translation of radiomics remains hindered by several challenges.^4^ Among these, the impact of CT reconstruction algorithms on image quality and consequently on the extraction and stability of radiomic features,^5^, ^6^ stands out as a critical technical confounder requiring rigorous investigation.

The evolution of CT reconstruction techniques, from traditional filtered back projection (FBP) to iterative reconstruction (IR)^7^ and more recently to deep learning‐based reconstruction (DLR)^8^, ^9^ algorithms, represents a paradigm shift in clinical imaging. Among iterative techniques, adaptive statistical iterative reconstruction‐Veo (ASIR‐V), a third‐generation hybrid iterative reconstruction method, employs both statistical modeling and system optical modeling to allow for radiation dose reduction while enhancing conventional image quality compared to FBP.^10^ However, IR can produce an unnatural “plastic‐like” appearance and may alter the image texture, particularly at higher iteration strengths.^11^, ^12^ In response to these limitations, state‐of‐the‐art DLR algorithms have emerged as a breakthrough.^13^, ^14^ A notable example is the deep learning image reconstruction (DLIR) algorithm, which utilizes a deep neural network trained on high‐quality FBP‐based images to reconstruct CT data and offers three levels (low, medium and high) of reconstruction strength tailored to varying noise reduction requirements. DLIR not only achieves greater radiation dose reduction and improved image quality compared to both IR and FBP,^15^, ^16^, ^17^, ^18^, ^19^, ^20^, ^21^, ^22^, ^23^ but it also preserves noise texture more akin to that of FBP.^24^, ^25^ Given the wide clinical application of DLIR algorithms, it is imperative to systematically investigate their impact on the radiomic workflow.

Previous studies have indeed highlighted the vulnerability of radiomic features to reconstruction algorithms.^26^, ^27^, ^28^, ^29^, ^30^ Some recent work has also suggested that DLIR can improve the robustness of radiomic features at different radiation dose levels.^31^, ^32^, ^33^ However, a significant gap persists in the literature. Most existing investigations typically focus on quantifying feature‐value changes or statistical differences across acquisition and reconstruction parameters. There has been limited exploration into whether such technical variability ultimately compromises the model performance and, crucially, the transferability of radiomics models when applied to specific clinical tasks—which is the ultimate measure of their practical utility. While the diagnostic benefits of DLIR for human readers are well established, its impact on the downstream radiomics pipeline, especially model generalizability across datasets derived from different reconstruction techniques, remains inadequately explored within a task‐based framework.^34^, ^35^ This is a critical omission because feature variability observed in isolation (a technical observation) has limited clinical relevance unless its impact is assessed against a clear clinical endpoint.

Therefore, bridging this gap, we hypothesized that although DLIR may introduce technique‐specific radiomic feature variability, this variability does not fundamentally undermine the model's ability to capture underlying biological information relevant to clinical decision‐making. To test this hypothesis, we conducted a task‐based assessment using AP severity stratification as the clinically relevant endpoint. Our study aimed to investigate the transfer performance of radiomics models across datasets derived from images reconstructed with DLIR, ASIR‐V, and FBP. Our core objective was to clarify whether DLIR algorithms affect the transferability of radiomics models, rather than to validate the utility of radiomics for AP severity stratification itself.

## 2 METHODS

### 2.1 Two advanced CT reconstruction algorithms

ASIR‐V is a commercially established hybrid iterative reconstruction algorithm, widely available across multiple CT scanner generations. It functions by iteratively minimizing the discrepancy between measured raw projection data and a forward‐projected estimate of the reconstructed image. This approach integrates statistical modeling to address quantum noise and system optics modeling to correct scanner‐specific hardware imperfections, such as detector response and focal spot blur. A user‐defined blending percentage (e.g., ASIR‐V 50%) determines the proportion of iterative reconstruction contribution combined with a baseline FBP reconstruction.

DLIR, commercially deployed as TrueFidelity by GE Healthcare, represents the latest‐generation deep learning‐based reconstruction method, available on newer generations of GE CT scanners (e.g., Revolution Apex and later models). It employs a deep convolutional neural network trained on an extensive dataset of high‐quality, low‐noise images derived from routine‐dose scans reconstructed via FBP. The network learns a direct mapping from noisy, low‐dose sinogram data or initial low‐fidelity reconstructions to high‐quality images. In contrast to the adjustable blending in ASIR‐V, DLIR operates at predefined strength levels (low, medium, and high) that are not simple linear increments but involve a fundamental trade‐off between denoising power and preserving natural image texture. Its principal technical advantage lies in achieving significant noise suppression and preservation of spatial resolution while maintaining a natural, FBP‐like noise texture, thereby avoiding the artificial visual artifacts commonly associated with high‐level iterative reconstruction techniques.

### 2.2 Patient cohort

**FIGURE 1:**
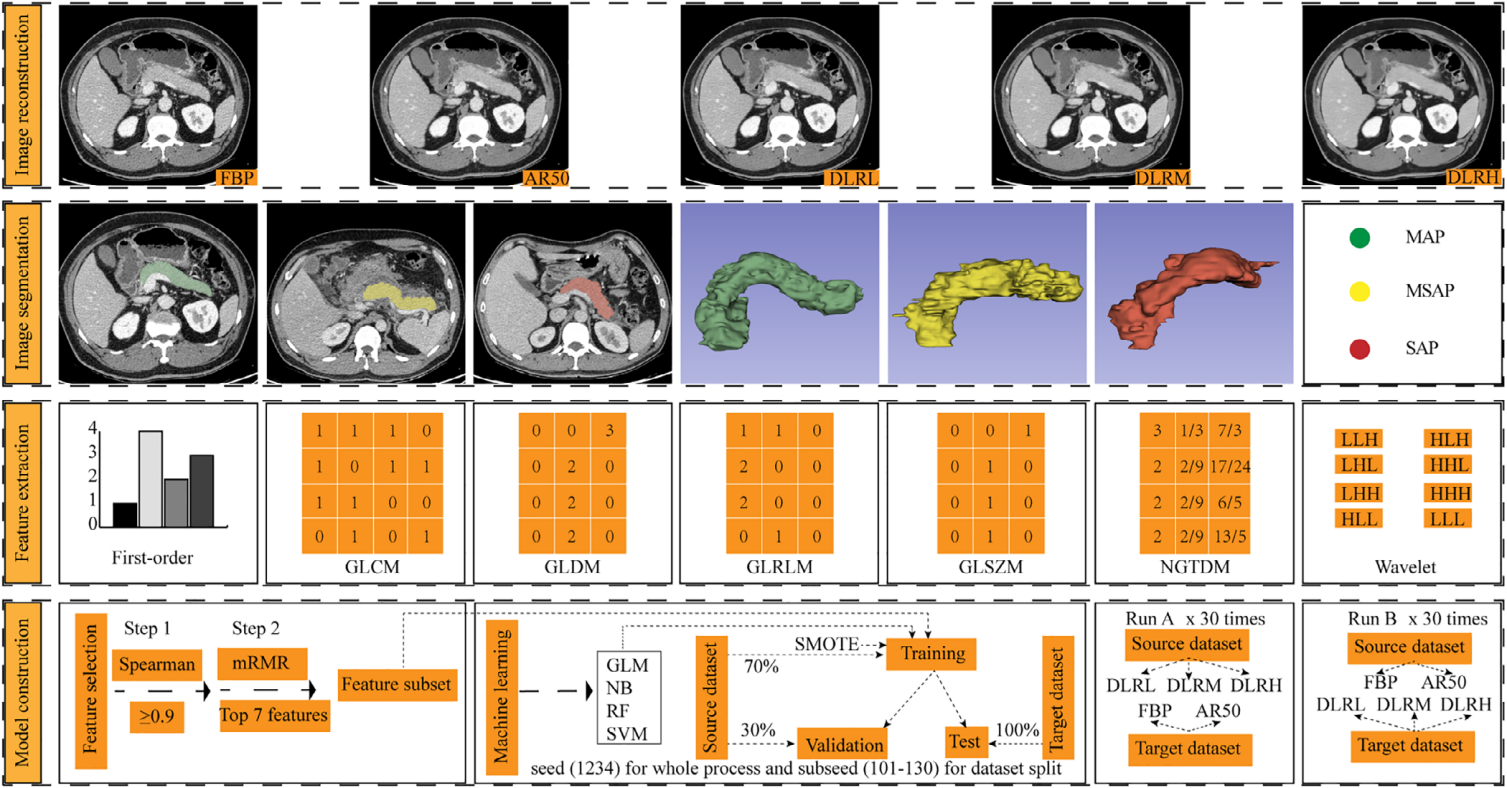
Workflow of the radiomics analysis for assessing model transferability across CT reconstruction algorithms. The radiomic analysis pipeline consists of four stages: (1) **Image reconstruction**: Five sets of portal venous phase images were reconstructed using filtered back projection (FBP), adaptive statistical iterative reconstruction‐Veo (ASIR‐V) at 50% blending level (AR50), and deep learning image reconstruction (DLIR) at low (DLRL), medium (DLRM), and high (DLRH) strengths. (2) **Image segmentation**: Volumes of interest were manually delineated on FBP axial images, covering the pancreatic parenchyma while excluding vessels, ducts, and necrotic tissue. (3) **Feature extraction**: A total of 837 radiomic features were extracted, including 18 first‐order features, 75 texture features (GLCM, GLDM, GLRLM, GLSZM, NGTDM) and 744 wavelet transform‐based features (93 features multiplying eight filter modes). (4) **Model construction**: The DLIR datasets (DLRL, DLRM and DLRH dataset) were interchanged with the AR50 and FBP datasets as the source and target datasets in two respective experimental configurations. The source dataset was partitioned into a training set and a validation set at a ratio of 70%/30%, while the target dataset was utilized as the test set. After feature selection via Spearman rank correlation test and mRMR, four machine learning classifiers—generalized linear model (GLM), naive Bayes (NB), random forest (RF), and support vector machine (SVM)—were trained on the training set with SMOTE to predict AP severity. Then the models were validated on the validation set and tested on the test set without changing the model parameters. Thirty randomized experiments were conducted from dataset partitioning and feature selection to model training and evaluation. GLCM = gray level co‐occurrence matrix, GLDM = gray level dependence matrix, GLRLM = gray level run length matrix, GLSZM = gray level size zone matrix, NGTDM = neighborhood gray tone difference matrix, mRMR = maximum relevance minimum redundancy, SMOTE = Synthetic Minority Over‐sampling Technique, AP = acute pancreatitis.

The study workflow is illustrated in Figure 1. Patients diagnosed with AP who underwent abdominal contrast‐enhanced CT scans between April and August 2024 were identified via the institutional picture archiving and communication system (PACS) and electronic medical records. The inclusion criteria were: (1) clinical and radiological confirmation of AP; (2) age ≥ 18 years; and (3) adequate image quality without metal artifacts near the pancreas. The exclusion criteria were: (1) incomplete clinical data; (2) non‐baseline CT scans (performed ≥ 2 weeks post‐admission, potentially failing to reflect initial AP severity); and (3) combined with pancreatic malignancy.

**FIGURE 2:**
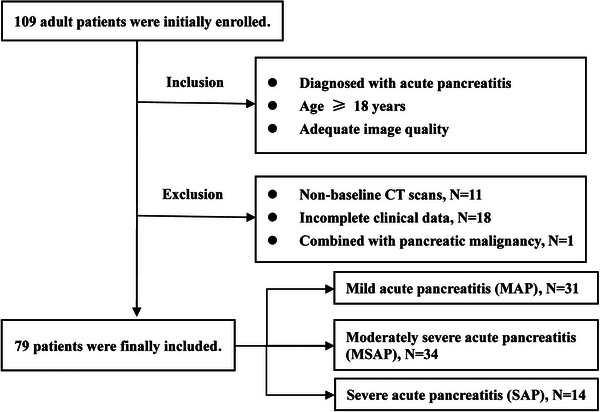
Flowchart of patient inclusion and exclusion.

The final cohort comprised 79 AP patients (54 men, 25 women) (Figure 2). Based on the revised Atlanta criteria,^36^ patients were categorized by AP severity as follows: 31 with mild AP (MAP), 34 with moderately severe AP (MSAP), and 14 with severe AP (SAP). Other cohort demographics were: mean (± standard deviation) age 44.89 ± 15.03 years, average height 166.10 ± 8.34 cm, and mean weight 71.09 ± 15.46 kg. Aligned with the study's primary objective of evaluating the impact of CT reconstruction algorithms on the performance of radiomics models, clinical variables were intentionally excluded from modeling to avoid confounding by clinical covariates.

### 2.3 CT acquisition and image reconstruction

All patients underwent scanning in the supine position during maximum inspiration using a 256‐row spiral CT scanner (Revolution CT, GE Healthcare). The contrast‐enhanced CT acquisition parameters were as follows: tube voltage, 120 kVp; tube current, 250–500 mA; slice thickness/spacing, 5 mm; pitch, 0.992:1; field of view, 300 × 400 mm; and matrix size, 512 × 512. The noise index was set to 9.7 under the primary reconstruction protocol, which employed a standard convolution kernel and a slice thickness of 5 mm. For contrast administration, 1.2 mL/kg of a nonionic contrast agent (Ioversol, 350 mgI/mL) was injected via the cubital vein at a flow rate of 3.0–4.0 mL/s. Portal venous phase imaging was performed 65–70 s after the initiation of contrast injection, in line with standard abdominal imaging protocols.

Scanning raw data from the portal venous phase were prospectively reconstructed on another CT scanner (Revolution Apex CT, GE Healthcare) using five distinct algorithms to generate comparative image sets: (1) conventional FBP; (2) ASIR‐V at 50% blending level (AR50, routine for clinical use at our institution); and (3) DLIR at low (DLRL), medium (DLRM), and high (DLRH) strengths. All reconstructed images were generated with a slice thickness and spacing of 1.25 mm and stored in anonymized DICOM format to comply with privacy protocols.

### 2.4 Image segmentation and radiomic feature extraction

DICOM images from all patients were imported into 3D Slicer (version 5.6.2, https://www.slicer.org) for segmentation and radiomic feature extraction. A radiologist with 5 years of experience in abdominal CT imaging manually delineated volumes of interest (VOIs) covering the pancreatic parenchyma exclusively on the FBP axial images. This single‐set (width, 350 HU; level, 40 HU) segmentation approach was adopted to ensure anatomical consistency across all five reconstruction methods, thereby isolating the effect of reconstruction algorithms from segmentation variability.

Radiomic features were extracted from the delineated VOIs via the open‐source Radiomics package integrated in 3D Slicer. Prior to feature extraction, VOIs were resampled to a uniform voxel size of 1 × 1 × 1 mm^3^, and intensity discretization was performed with a fixed bin number of 25. To ensure comparability across reconstruction techniques, the same VOI delineations were uniformly applied for feature extraction across all five reconstruction groups.

A total of 837 radiomics features were extracted from the original image as well as from various filtered versions for each patient's five reconstructed image sets, categorized as follows: (1) 18 first‐order features (intensity‐based metrics, e.g., mean, entropy); (2) 75 texture features from gray‐level matrices, including gray level co‐occurrence matrix (GLCM), gray level dependence matrix (GLDM), gray level run length matrix (GLRLM), gray level size zone matrix (GLSZM), and neighborhood gray tone difference matrix (NGTDM); and (3) 744 wavelet transform features (93 features × 8 filter modes: LLH, LHL, LHH, HLL, HLH, HHL, HHH, and LLL) using the Coiflet 1 (coif1) wavelet basis function, a commonly used choice in medical image processing. Morphological features were excluded, as segmented VOIs were identical across all reconstruction methods to preclude potential variability in these metrics. All first‐order and texture features were listed in Supplementary Table S1.

### 2.5 Analysis of differences in features

Our analytical strategy was designed to quantify the disparities in radiomic features arising from different reconstruction algorithms, ranging from the global feature space to localized key features. To characterize feature‐level variations, we began with a global assessment. All features within each source‐target dataset pair were first standardized via Z‐score normalization. We then employed Principal Component Analysis (PCA) to visually inspect the overall separability of feature spaces between reconstruction methods, followed by a Permutational Multivariate Analysis of Variance (PERMANOVA) with 1000 permutations to statistically test for significant global divergence. Subsequently, we conducted univariate analyses to all individual features. The practical significance of differences between individual features was measured using Cohen's d, with effect sizes categorized as trivial (|d| < 0.2), small (0.2 ≤ |d| < 0.5), medium (0.5 ≤ |d| < 0.8), or large (|d| ≥ 0.8).

### 2.6 Feature selection and model development

All modeling procedures were implemented in R (version 4.4.1), and the R packages and key hyperparameters involved in modeling are shown in Supplementary Table S2. The bidirectional transferability of radiomics models was assessed in two experimental configurations where DLIR datasets (DLRL, DLRM, DLRH) were alternately paired with AR50 or FBP datasets as the source and target, respectively. The source dataset was partitioned into training (70%) and validation (30%) sets using stratified sampling to preserve the class distribution. To ensure reproducibility and mitigate bias from a single random split, this partitioning process was repeated 30 times using definite random seeds (101 to 130). The corresponding target dataset was held out as an independent test set.

A two‐stage feature selection pipeline was applied independently within each of the 30 training sets to prevent data leakage and ensure robust feature identification. First, to mitigate multicollinearity, we calculated Spearman's rank correlation coefficient. For any pair of features with an absolute correlation coefficient > 0.9, one feature was randomly discarded. Second, the remaining features were ranked using the maximum relevance minimum redundancy (mRMR) algorithm.^37^ The optimal number of features was fixed at 7 across all prediction models, which was determined via 5‐fold cross‐validation on the training data, selecting the count that yielded the highest mean AUC (area under the receiver operating characteristic curve) with the lowest standard deviation (SD). In addition, to investigate the underlying causes of variations in model performance, we ranked the selected features based on their frequency across 30 randomized experiments and focused on the top 15 features, which collectively accounted for over 50% of the total occurrences. Then, the resulting optimal feature subset was standardized using Z‐score normalization (mean = 0, SD = 1).

A binary classification model was constructed using the optimal feature subset to distinguish mild cases (MAP) from severe cases (MSAP and SAP). To address class imbalance in the training data, the Synthetic Minority Over‐sampling Technique (SMOTE) was applied. Four distinct machine learning algorithms were employed: Generalized Linear Model (GLM), Naive Bayes (NB), Random Forest (RF), and Support Vector Machine (SVM). To establish a baseline performance comparison across these algorithmic approaches, models were constructed using their default hyperparameters as implemented in the respective R packages. Each trained model was then evaluated on its corresponding validation set and, critically, on the independent test set to assess its transferability. For overall experimental reproducibility, while the data partitions were generated with varying seeds, all other stochastic processes (e.g., SMOTE, model initialization) were governed by a single fixed random seed (1234).

### 2.7 Statistical analysis

All statistical analyses were conducted in R (version 4.4.1; https://www.r‐project.org/) within RStudio (version 2024.04.2+764; https://www.rstudio.com/), utilizing relevant extensions as indicated in the R handbook.^38^ The Shapiro–Wilk test was used to assess data normality, guiding the choice between an independent *t*‐test and a Wilcoxon rank‐sum test for comparing single feature difference.

For model performance evaluation, the area under the receiver operating characteristic curve (AUC), accuracy, sensitivity and specificity were calculated on the validation set and the test set across the 30 independent runs, reporting the mean and 95% confidence interval (CI) for each metric. AUC was designated as the primary performance endpoint due to its comprehensive measure of discriminatory power of models. A key metric for our study, termed the “transfer performance drop” (ΔAUC) was defined as: ΔAUC = AUC_validation (source)—AUC_test (target). A positive ΔAUC indicates a performance drop upon transfer. We used a paired t‐test to assess the statistical significance of this drop for each model and transfer scenario. To compare the relative robustness of different machine learning algorithms, independent t‐tests were employed to analyze differences in the magnitude of the transfer performance drop between model pairs. To account for multiple comparisons across all statistical tests, *P* values were adjusted using the False Discovery Rate (FDR) correction. An adjusted *P* value under 0.05 was considered statistically significant.

## 3 RESULTS

### 3.1 Feature divergence across reconstruction algorithms

**FIGURE 3:**
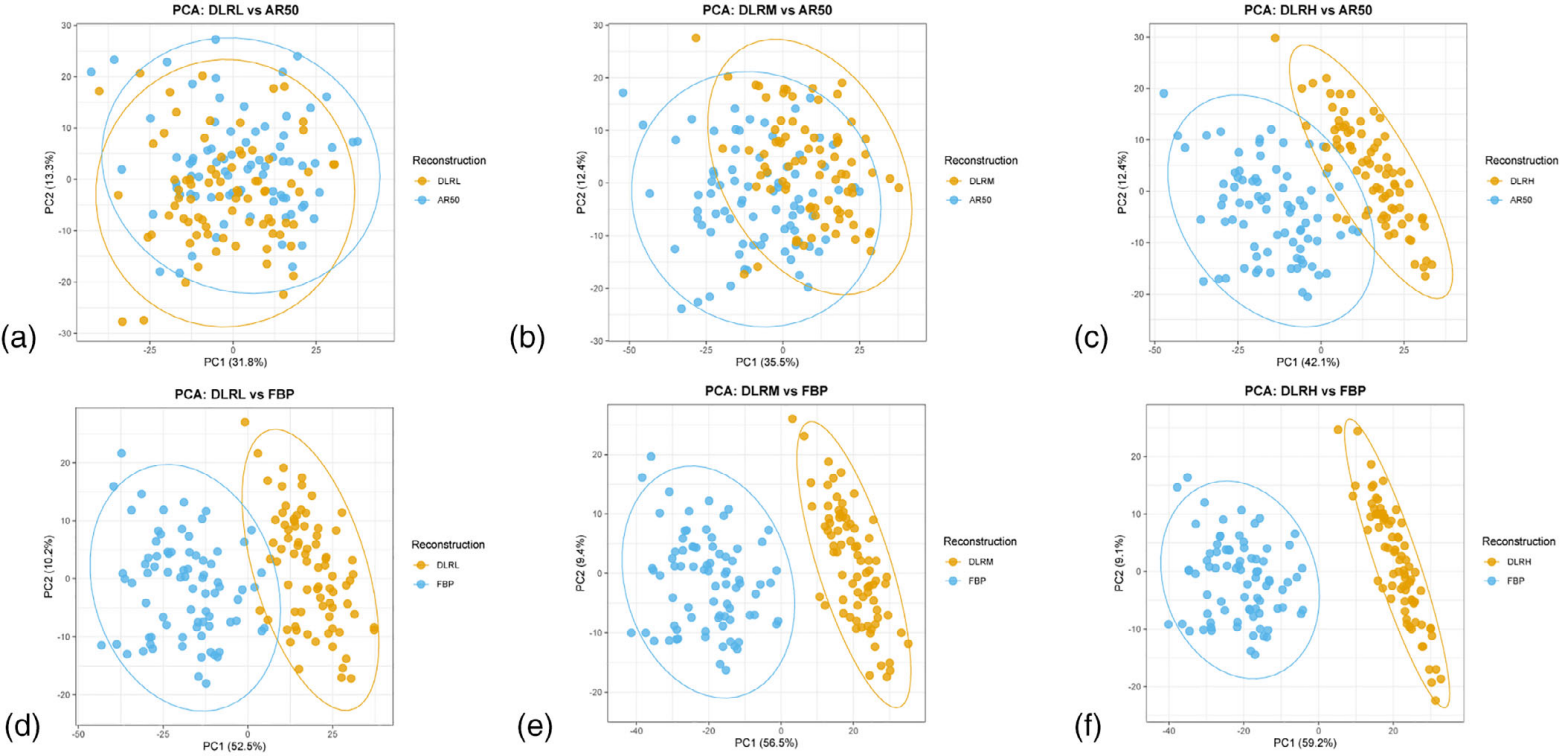
Progressive divergence of radiomic feature space with increasing strength of deep learning image reconstruction (DLIR). (a–f) Principal component analysis (PCA) was conducted to assess the separation of global feature space between the paired datasets, based on all 837 radiomic features initially extracted. The x‐axis represents the first principal component (PC1), and the y‐axis represents the second principal component (PC2); the percentages in parentheses denote the proportion of total variance explained by each respective component. FBP = filtered back projection; ASIR‐V = adaptive statistical iterative reconstruction‐Veo; AR50 = ASIR‐V at 50% blending level; DLRL/DLRM/DLRH = DLIR at low/medium/high strength.

The divergence of radiomic features between DLIR and conventional reconstruction algorithms intensified with increasing the strength of DLIR (Figure 3 and Supplementary Table S3). When compared with AR50, the number of significantly different features increased from 279 (DLRL) to 719 (DLRH), and the mean absolute effect size rose from 0.314 to 1.323. This divergence was even more pronounced relative to FBP, where the count of significantly different features was already high at the lowest DLIR strength (756) and increased further to 777 (DLRH), with the mean absolute effect size escalating from 1.863 to 2.940. Accordingly, the proportion of features with moderate‐to‐large effect sizes (|d| ≥ 0.5) expanded from 182 to 631 against AR50 and from 700 to 733 against FBP.

In line with these, multivariate analysis using PERMANOVA corroborated the univariate trends, revealing a significant global separation in the feature space between DLRH and AR50 (*P* < 0.05), as well as between all DLIR levels and FBP (*P* < 0.05). In contrast, no significant global separation was detected between the DLRL/DLRM and AR50 datasets (*P* > 0.05).

### 3.2 Stability analysis of frequently selected features

The stability of the 15 most frequently selected features (accounting for over 50% of total selections) from each of the five datasets was analyzed, and it varied by reconstruction algorithms (Supplementary Table S4). As DLIR strength increased, features co‐selected by DLIR and AR50 decreased from 9 (DLRL) to 3 (DLRH), while those shared between DLIR and FBP dropped from 5 to 2. However, models trained on DLIR datasets generally achieved higher AUC values in the validation set compared to AR50 and FBP, particularly at higher DLIR strengths, despite no statistical differences for almost all pairwise comparisons (Table 1). Additionally, for features selected from DLIR datasets, the number of those with small effect‐size difference (|d| < 0.5) decreased from 13 (DLRL) to 4 (DLRH) compared to AR50, and from 5 to 2 compared to FBP. In contrast, for features selected from AR50 and FBP datasets, the corresponding numbers were 11, 8, and 6 for AR50 and 8, 5, and 8 for FBP when compared to DLRL, DLRM, and DLRH, respectively.

**TABLE 1:** Model performance comparison between DLIR of different intensities and AR50 and FBP in the validation set.

Model	DLRL (mean AUC with 95%CI)	FBP (mean AUC with 95%CI)	*P* value	AR50 (mean AUC with 95%CI)	*P* value
GLM	0.715 (0.684–0.746)	0.707 (0.669–0.745)	0.954	0.725 (0.691–0.760)	0.871
NB	0.754 (0.722–0.786)	0.748 (0.713–0.783)	0.954	0.774 (0.745–0.803)	0.689
RF	0.792 (0.770–0.815)	0.740 (0.712–0.768)	0.017	0.775 (0.751–0.799)	0.689
SVM	0.725 (0.692–0.758)	0.724 (0.691–0.756)	0.954	0.726 (0.691–0.762)	0.946

Model	DLRM (mean AUC with 95%CI)	FBP (mean AUC with 95%CI)	*P* value	AR50 (mean AUC with 95%CI)	*P* value
GLM	0.755 (0.715–0.796)	0.707 (0.669–0.745)	0.108	0.725 (0.691–0.760)	0.256
NB	0.810 (0.781–0.840)	0.748 (0.713–0.783)	0.014	0.774 (0.745–0.803)	0.162
RF	0.806 (0.779–0.834)	0.740 (0.712–0.768)	0.004	0.775 (0.751–0.799)	0.162
SVM	0.759 (0.722–0.796)	0.724 (0.691–0.756)	0.145	0.726 (0.691–0.762)	0.256

Model	DLRH (mean AUC with 95%CI)	FBP (mean AUC with 95%CI)	*P* value	AR50 (mean AUC with 95%CI)	*P* value
GLM	0.765 (0.732–0.798)	0.707 (0.669–0.745)	0.028	0.725 (0.691–0.760)	0.231
NB	0.802 (0.773–0.830)	0.748 (0.713–0.783)	0.028	0.774 (0.745–0.803)	0.231
RF	0.793 (0.768–0.818)	0.740 (0.712–0.768)	0.021	0.775 (0.751–0.799)	0.278
SVM	0.765 (0.724–0.806)	0.724 (0.691–0.756)	0.116	0.726 (0.691–0.762)	0.231

*Note*: *P* value indicates the results of comparisons using independent *t*‐test for AUC of different machine learning models between DLIR (DLRL, DLRM, DLRH) and FBP as well as between DLIR and AR50, and were adjusted using the False Discovery Rate (FDR) correction.

Abbreviations: AR50, ASIR‐V at 50% blending level; ASIR‐V, adaptive statistical iterative reconstruction‐Veo; AUC, area under the receiver operating characteristic curve; CI, confidence interval; DLIR, deep learning image reconstruction; DLRL/DLRM/DLRH, DLIR at low/medium/high strength; FBP, filtered back projection; GLM, generalized linear model; NB, Naive Bayes; RF, random forest; SVM, support vector machine.

Notably, four features were highly stable (no significant differences across all five reconstruction methods): *wavelet_LHL_firstorder_Mean*, *wavelet_HHH_firstorder_Skewness*, *wavelet_HLH_firstorder_Mean*, and *wavelet_LLL_glszm_SmallAreaEmphasis*. Their selection frequency among the top 15 selected features varied by algorithms: all 4 for FBP, 1 for AR50, and 2, 1, 0 for DLRL, DLRM, and DLRH, respectively.

### 3.3 Transferability of models across reconstruction algorithms

**FIGURE 4:**
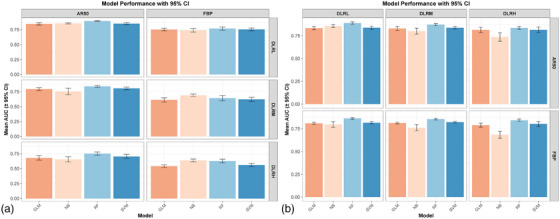
Asymmetric model performance (mean AUC and 95% CI) of four machine learning models in the bidirectional transfer experiments. (a) The transfer direction from DLIR (DLRL, DLRM, DLRH) datasets to AR50 and FBP datasets; (b) The transfer direction from AR50 and FBP datasets to DLIR datasets. AUC = area under the receiver operating characteristic curve; CI = confidence interval; DLIR = deep learning image reconstruction; FBP = filtered back projection; ASIR‐V = adaptive statistical iterative reconstruction‐Veo; AR50 = ASIR‐V at 50% blending level; DLRL/DLRM/DLRH = DLIR at low/medium/high strength; GLM = Generalized Linear Model; NB = Naive Bayes; RF = Random Forest; SVM = Support Vector Machine.

**FIGURE 5:**
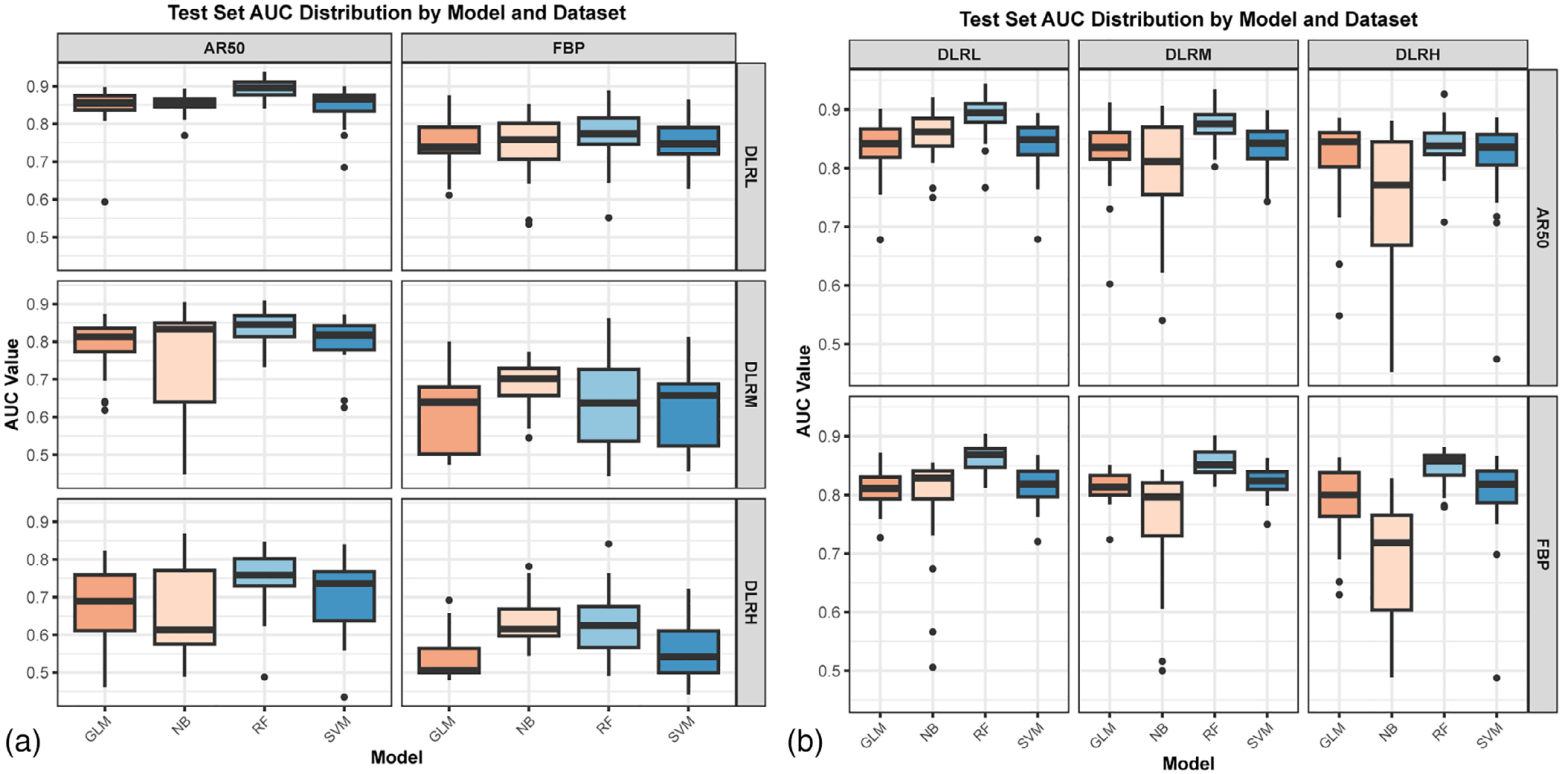
Distribution of model performance (median AUC and interquartile range) of four machine learning models in 30 random experiments on target datasets in two transfer directions. (a) The transfer direction from DLIR (DLRL, DLRM, DLRH) datasets to AR50 and FBP datasets; (b) The transfer direction from AR50 and FBP datasets to DLIR datasets. AUC = area under the receiver operating characteristic curve; CI = confidence interval; DLIR = deep learning image reconstruction; FBP = filtered back projection; ASIR‐V = adaptive statistical iterative reconstruction‐Veo; AR50 = ASIR‐V at 50% blending level; DLRL/DLRM/DLRH = DLIR at low/medium/high strength; GLM = Generalized Linear Model; NB = Naive Bayes; RF = Random Forest; SVM = Support Vector Machine.

**FIGURE 6:**
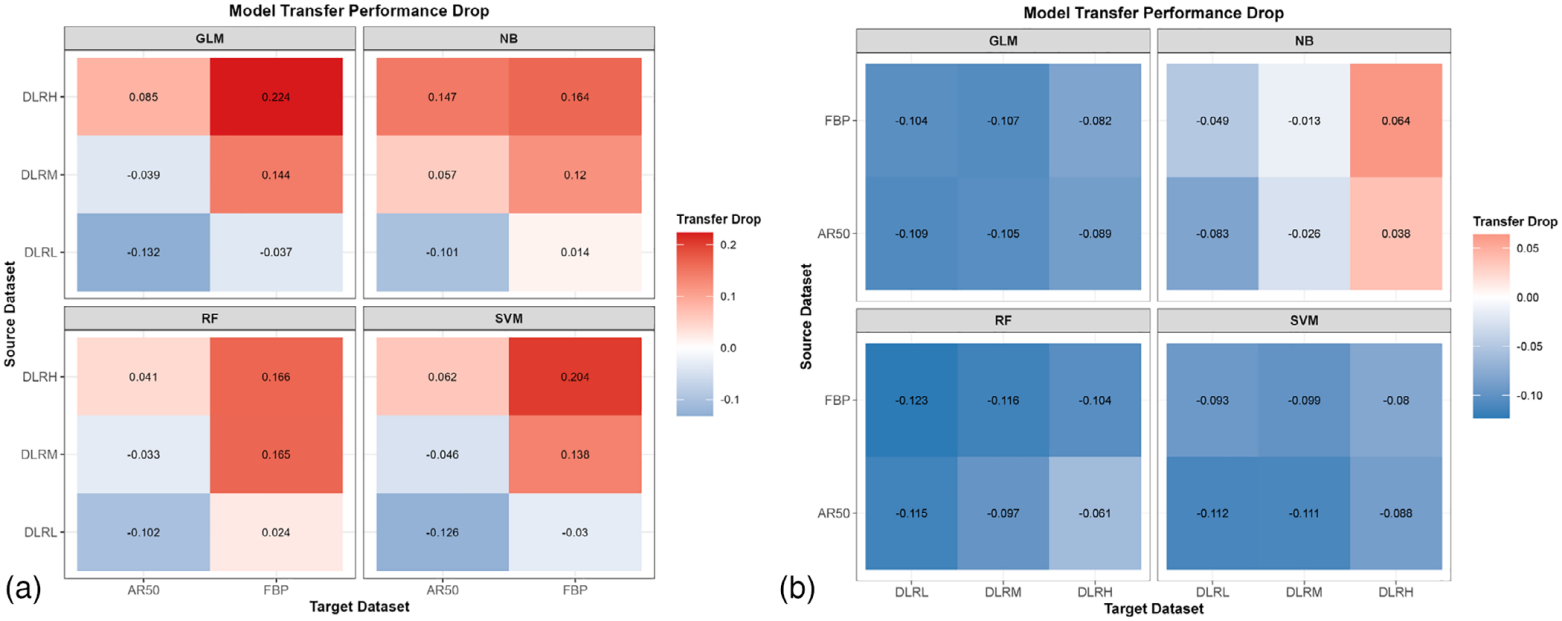
Quantification of the asymmetric transfer performance drop (ΔAUC) of four machine learning models in two transfer directions. (a) The transfer direction from DLIR (DLRL, DLRM, DLRH) datasets to AR50 and FBP datasets; (b) The transfer direction from AR50 and FBP datasets to DLIR datasets. AUC = area under the receiver operating characteristic curve; CI = confidence interval; DLIR = deep learning image reconstruction; FBP = filtered back projection; ASIR‐V = adaptive statistical iterative reconstruction‐Veo; AR50 = ASIR‐V at 50% blending level; DLRL/DLRM/DLRH = DLIR at low/medium/high strength; GLM = Generalized Linear Model; NB = Naive Bayes; RF = Random Forest; SVM = Support Vector Machine.

The transferability of radiomics models was asymmetric and dependent on both the source dataset and DLIR strength. When models trained on DLIR datasets were transferred to AR50 or FBP target datasets, their performance was inconsistent (Figure 4a). The average AUC on target datasets decreased progressively with increasing DLIR strength, with a more pronounced decline observed on FBP dataset compared to AR50. The distribution of AUC values across 30 randomized experiments indicated that the DLRL‐based model demonstrated the highest transferability (Figure 5a). Specifically, model transfers from DLRM to FBP and from DLRH to AR50 or FBP resulted in significant reductions in AUC (*P* < 0.05), with a maximum decrease of 0.224 (*P* < 0.001) (Figure 6a and Table 2). In other migration scenarios, changes in mean AUC were non‐inferior, with a maximum reduction of 0.057 (*P* = 0.081) and a maximum increase of 0.132 (*P* < 0.001).

**TABLE 2:** The transfer performance of models from deep learning image reconstruction (DLIR) datasets to conventional (AR50/FBP) datasets.

Model	Source dataset	Target dataset	AUC_validation_, (mean, 95%CI)	AUC_test_, (mean, 95%CI)	AUC_validation_‐AUC_test_	*P* value
GLM	DLRL	AR50	0.715 (0.684–0.746)	0.847 (0.827–0.867)	−0.132	< 0.001
		FBP	0.715 (0.684–0.746)	0.752 (0.729–0.775)	−0.037	0.021
	DLRM	AR50	0.755 (0.715–0.796)	0.794 (0.769–0.818)	−0.039	0.079
		FBP	0.755 (0.715–0.796)	0.612 (0.576–0.647)	0.144	< 0.001
	DLRH	AR50	0.765 (0.732–0.798)	0.680 (0.642–0.719)	0.085	0.001
		FBP	0.765 (0.732–0.798)	0.541 (0.519–0.564)	0.224	< 0.001
NB	DLRL	AR50	0.754 (0.722–0.786)	0.855 (0.846–0.865)	−0.101	< 0.001
		FBP	0.754 (0.722–0.786)	0.741 (0.711–0.770)	0.014	0.531
	DLRM	AR50	0.810 (0.781–0.840)	0.754 (0.699–0.808)	0.057	0.081
		FBP	0.810 (0.781–0.840)	0.690 (0.669–0.711)	0.120	< 0.001
	DLRH	AR50	0.802 (0.773–0.830)	0.655 (0.612–0.698)	0.147	< 0.001
		FBP	0.802 (0.773–0.830)	0.638 (0.615–0.661)	0.164	< 0.001
RF	DLRL	AR50	0.792 (0.770–0.815)	0.895 (0.885–0.904)	−0.102	< 0.001
		FBP	0.792 (0.770–0.815)	0.768 (0.742–0.795)	0.024	0.114
	DLRM	AR50	0.806 (0.779–0.834)	0.839 (0.823–0.855)	−0.033	0.075
		FBP	0.806 (0.779–0.834)	0.641 (0.598–0.684)	0.165	< 0.001
	DLRH	AR50	0.793 (0.768–0.818)	0.752 (0.725–0.779)	0.041	0.041
		FBP	0.793 (0.768–0.818)	0.627 (0.596–0.659)	0.166	< 0.001
SVM	DLRL	AR50	0.725 (0.692–0.758)	0.851 (0.835–0.867)	−0.126	< 0.001
		FBP	0.725 (0.692–0.758)	0.755 (0.732–0.777)	−0.030	0.074
	DLRM	AR50	0.759 (0.722–0.796)	0.805 (0.785–0.826)	−0.046	0.034
		FBP	0.759 (0.722–0.796)	0.621 (0.587–0.656)	0.138	< 0.001
	DLRH	AR50	0.765 (0.724–0.806)	0.702 (0.667–0.738)	0.062	0.034
		FBP	0.765 (0.724–0.806)	0.560 (0.533–0.587)	0.204	< 0.001

*Note*: *P* value indicates the results of comparisons using paired *t*‐test for AUC of the validation set and the test set for each paired transfer from DLIR (DLRL, DLRM, DLRH) to AR50 and FBP datasets, and *P* values were adjusted using the False Discovery Rate (FDR) correction.

Abbreviations: AR50, ASIR‐V at 50% blending leve; ASIR‐V, adaptive statistical iterative reconstruction‐Veol; AUC, area under the receiver operating characteristic curve; CI, confidence interval; DLIR, deep learning image reconstruction; DLRL/DLRM/DLRH, DLIR at low/medium/high strength; FBP, filtered back projection; GLM, generalized linear model; NB, Naive Bayes; RF, random forest; SVM, support vector machine.

Inversely, models trained on AR50 or FBP datasets demonstrated robust performance when transferred to DLIR datasets. Except for the NB model, other models maintained stable AUC values across all DLIR strength levels (Figure 4b), and the AUC distribution was more concentrated (Figure 5b). For GLM, RF, and SVM models, transfer to DLIR datasets yielded general improvements in performance (*P* < 0.001), with mean AUC increases ranging from 0.061 to 0.123 (*P* < 0.001) (Figure 6b and Table 3). The NB model showed a performance decline only when transferred from FBP to DLRH (ΔAUC = 0.064, *P* = 0.011), while performing comparably in all other cases.

**TABLE 3:** The transfer performance of models from conventional (AR50/FBP) datasets to deep learning image reconstruction (DLIR) datasets.

Model	Source dataset	Target dataset	AUC_validation_, (mean, 95%CI)	AUC_test_, (mean, 95%CI)	AUC_validation_‐AUC_test_	*P* value
GLM	AR50	DLRL	0.725 (0.691–0.760)	0.834 (0.817–0.851)	−0.109	< 0.001
	AR50	DLRM	0.725 (0.691–0.760)	0.830 (0.808–0.852)	−0.105	< 0.001
	AR50	DLRH	0.725 (0.691–0.760)	0.814 (0.786–0.842)	−0.089	< 0.001
	FBP	DLRL	0.707 (0.669–0.745)	0.810 (0.799–0.822)	−0.104	< 0.001
	FBP	DLRM	0.707 (0.669–0.745)	0.813 (0.804–0.823)	−0.107	< 0.001
	FBP	DLRH	0.707 (0.669–0.745)	0.789 (0.767–0.811)	−0.082	0.001
NB	AR50	DLRL	0.774 (0.745–0.803)	0.857 (0.841–0.872)	−0.083	< 0.001
	AR50	DLRM	0.774 (0.745–0.803)	0.801 (0.768–0.833)	−0.026	0.206
	AR50	DLRH	0.774 (0.745–0.803)	0.736 (0.689–0.783)	0.038	0.198
	FBP	DLRL	0.748 (0.713–0.783)	0.797 (0.766–0.827)	−0.049	0.024
	FBP	DLRM	0.748 (0.713–0.783)	0.761 (0.727–0.795)	−0.013	0.518
	FBP	DLRH	0.748 (0.713–0.783)	0.684 (0.646–0.722)	0.064	0.011
RF	AR50	DLRL	0.775 (0.751–0.799)	0.890 (0.878–0.903)	−0.115	< 0.001
	AR50	DLRM	0.775 (0.751–0.799)	0.872 (0.861–0.883)	−0.097	< 0.001
	AR50	DLRH	0.775 (0.751–0.799)	0.836 (0.821–0.851)	−0.061	< 0.001
	FBP	DLRL	0.740 (0.712–0.768)	0.863 (0.854–0.872)	−0.123	< 0.001
	FBP	DLRM	0.740 (0.712–0.768)	0.856 (0.847–0.865)	−0.116	< 0.001
	FBP	DLRH	0.740 (0.712–0.768)	0.844 (0.832–0.857)	−0.104	< 0.001
SVM	AR50	DLRL	0.726 (0.691–0.762)	0.838 (0.822–0.855)	−0.112	< 0.001
	AR50	DLRM	0.726 (0.691–0.762)	0.838 (0.824–0.851)	−0.111	< 0.001
	AR50	DLRH	0.726 (0.691–0.762)	0.815 (0.785–0.845)	−0.088	< 0.001
	FBP	DLRL	0.724 (0.691–0.756)	0.817 (0.805–0.829)	−0.093	< 0.001
	FBP	DLRM	0.724 (0.691–0.756)	0.823 (0.813–0.832)	−0.099	< 0.001
	FBP	DLRH	0.724 (0.691–0.756)	0.803 (0.777–0.830)	−0.080	< 0.001

*Note*: *P* value indicates the results of comparisons using paired *t*‐test for AUC of the validation set and the test set for each paired transfer from AR50 and FBP to DLIR (DLRL, DLRM, DLRH) datasets, and *P* values were adjusted using the False Discovery Rate (FDR) correction.

Abbreviations: AR50, ASIR‐V at 50% blending level; ASIR‐V, adaptive statistical iterative reconstruction‐Veo; AUC, area under the receiver operating characteristic curve; CI, confidence interval; DLIR, deep learning image reconstruction; DLRL/DLRM/DLRH, DLIR at low/medium/high strength; FBP, filtered back projection; GLM, generalized linear model; NB, Naive Bayes; RF, random forest; SVM, support vector machine.

Overall, the magnitude of AUC change did not differ among machine learning models when transferring from DLIR to AR50/FBP or vice versa (*P* > 0.05), except for several comparisons involving the NB model (Supplementary Table S5 and S6).

## 4 DISCUSSION

In this study, we systematically evaluated the bidirectional transferability of radiomics models across conventional (FBP, AR50) and deep learning‐based (DLIR) CT reconstruction algorithms, using the clinically relevant task of AP severity stratification. Our primary finding revealed a fundamental asymmetry in model generalizability: models trained on conventional reconstructions demonstrated robust performance when transferred to DLIR datasets, whereas the reverse was successful only when migrating from low‐strength DLIR. This nuanced finding underscores that the impact of DLIR on radiomics is not a simple matter of improvement or degradation, but is critically modulated by algorithm strength and transfer direction.

The observed asymmetric transferability can be traced back to the fundamental impact of reconstruction algorithms on radiomic features and the subsequent feature selection process. Consistent with prior work,^26^, ^28^ we observed that increasing DLIR strength amplified the statistical difference of radiomic features between DLIR and conventional reconstruction algorithms, particularly FBP. Our multivariate PERMANOVA analysis offered a more holistic picture: the global feature space distribution of low‐ to medium‐strength DLIR remained comparable to that of AR50, whereas DLIR of any strength induced a significant distributional shift in comparison with FBP. This phenomenon can be attributed to the nonlinear noise reduction mechanism inherent in DLIR. High‐strength DLIR, in particular, alters the image's noise texture by shifting the peak of the noise power spectrum (NPS) toward lower spatial frequencies, which is different from the structured, high‐frequency noise profile of FBP.^24^, ^25^, ^39^ Additionally, a significant portion of the reproducible features in FBP is considered to be driven by repetitive noise patterns.^31^ The significant noise reduction of DLIR would therefore suppress these noise‐dependent features, contributing to the observed feature space divergence. Instead, the closer alignment between low‐strength DLIR and AR50 may stem from their more comparable noise magnitude ratios (NMR), indicating an analogous image texture.^24^, ^40^

Crucially, these feature‐level shifts directly influenced which features were selected for the predictive models. When trained on feature datasets extracted from DLIR reconstructed images, the models likely learned to leverage highly refined, “algorithm‐specific” features that offered superior classification power within the low‐noise domain. However, these features proved to be non‐robust and lost their discriminative advantage when tested on datasets derived from conventional images with higher‐noise levels, leading to a performance collapse. A notable observation was that the model demonstrates improved performance on the AR50 dataset when DLRL was used as the source dataset. This improvement can be primarily attributed to the similarity in the spatial distribution of global features between the two datasets. Conversely, models trained on FBP or AR50 were forced to identify features robust against inherent image noise by selection, such as the four wavelet features frequently selected in the FBP dataset (e.g., *wavelet_LHL_firstorder_Mean*), the original features of which have also been reported stable across reconstruction algorithms in other literature.^26^, ^27^, ^28^, ^31^ These “algorithm‐agnostic” features not only provided a stable foundation for the model but, as our results suggest, may have their underlying biological signal amplified by the noise reduction in DLIR images,^31^, ^41^ explaining the observed performance boost upon the corresponding transfer.

Our findings enter a dialogue with a limited but growing body of literature. The general observation from prior studies is that overall diagnostic performance can be preserved across different reconstruction algorithms.^26^, ^34^ For instance, studies on cardiac mass discrimination^26^ and pulmonary nodule classification^34^ have reported that while the underlying difference in certain radiomic features, the ultimate classification performance of radiomics models remains comparable between deep learning and conventional reconstruction algorithms. While our results align with this observation of preserved performance, they also uncover a critical layer of complexity that previous work has not fully explored. First, by comprehensively evaluating all DLIR strength levels, we demonstrate that the reconstruction strength is a pivotal variable that affects feature stability and modeling process. Second, our bidirectional transferability assessment reveals a crucial performance asymmetry—a finding obscured in single‐direction studies—that has profound implications for model deployment in clinically heterogeneous datasets. Finally, the rigorous and high‐repeatability approach employed here ensures the statistical robustness of these nuanced findings and offers a methodological reference for future validation studies in this domain.

These discoveries yield critical insights for the field. Theoretically, we argue that DLIR strength must be treated as a fundamental experimental parameter, as critical to report and control as slice thickness or radiation dose. Practically, our work provides potentially actionable guidance: to build broadly generalizable models, training could be considered to perform on FBP or low‐strength DLIR data. Conversely, extreme caution is warranted when deploying models trained on high‐strength DLIR to legacy data or data from different systems. Methodologically, our study serves as a stark reminder that reconstruction algorithms must be considered a primary confounding variable in any radiomics investigation, demanding careful consideration in both study design and data harmonization.

This study has several limitations that should be acknowledged. As a single‐center, retrospective analysis using equipment from one vendor (GE Healthcare), the generalizability of our conclusions to other DLR algorithms and broader patient populations requires external validation. Furthermore, our investigation was confined to a single clinical task—AP severity stratification. The stability and relevance of radiomic features are task‐dependent, and future work should explore whether these findings hold for other applications, such as tumor classification or treatment response prediction. In addition, the exclusion of peripancreatic regions may compromise the generalizability of radiomics models for predicting AP severity in clinical settings. Future studies could explore the combined impact of multi‐region ROI definitions on model performance. Finally, our reliance on manual segmentation, while ensuring consistency, differs from the trend toward automated segmentation. The interplay between DLIR's texture modification and the performance of deep learning‐based segmentation tools represents a critical and unexplored frontier.

Future research should prioritize multi‐center, multi‐vendor studies to establish the broader validity of this asymmetric transferability. An exciting avenue would be to investigate the biological underpinnings of the algorithm‐agnostic features we identified (e.g., by correlating them with histopathological markers), moving beyond statistical stability to biological plausibility. Integrating automated segmentation into the workflow will also be essential for translating these findings into clinically feasible pipelines. Moreover, investigating the transferability of radiomics models between iterative reconstruction techniques—across various strength levels—and FBP constitutes a meaningful extension of the current study, with the potential to provide more comprehensive guidance for clinical radiomics workflows that rely on conventional reconstruction algorithms.

In conclusion, this task‐based assessment demonstrates that the impact of DLIR on radiomics is nuanced, modulated by both algorithm strength and transfer direction. While DLIR does not fundamentally compromise the diagnostic performance for AP severity stratification, it creates an asymmetric transfer landscape: models readily transfer from conventional reconstructions to DLIR, but the reverse transfer is only successful from low‐strength DLIR. These findings underscore the need for a deliberate reconstruction strategy in radiomics and provide preliminary insights for developing robust, generalizable models in an era of deep learning‐based CT reconstruction algorithms.

## AUTHOR CONTRIBUTIONS

(I) Conception and design: Xiaobao Hu, Mengya Guo, Fuqing Zhou; (II) Administrative support: Zhihua Wu, Fuqing Zhou; (III) Provision of study materials or patients: Zhongren Huang, Zhihua Wu; (IV) Collection and assembly of data: Xiaobao Hu, Zhongren Huang; (V) Data analysis and interpretation: Xiaobao Hu, Xiao Liang, Yanyan Zhu, Mengya Guo; (VI) Manuscript writing: All authors; (VII) Final approval of manuscript: All authors.

## CONFLICT OF INTEREST STATEMENT

The authors have no conflict of interest to disclose.

## ETHICS STATEMENT

This retrospective study was approved by the Medical Research Ethics Committee of the First Affiliated Hospital of Nanchang University (No. 2024760), and the requirement for written informed consent was waived in accordance with institutional guidelines for retrospective studies involving anonymized data.

## Supporting information



Supporting information
