# Supplementary material for: Transferability of radiomics models between deep learning and conventional CT reconstruction algorithms: A task‐based assessment for stratifying acute pancreatitis severity
Source: J Appl Clin Med Phys. 2026 Mar 25;27(4):e70551. doi: 10.1002/acm2.70551 (PMC13140197; doi:10.1002/acm2.70551)
Supplement: Supplementary file 1 — Supporting information [file ACM2-27-e70551-s001.pdf]

Supplementary Material

SUPPLEMENTARY TABLE S1 The specific R packages and key hyperparameters for modeling

Model	R package (version)	key hyperparameters
GLM	Stats 4.4.1	Family = binomial, link = logit
NB	klaR 1.7.3	Laplace smoothing parameter = 0, usekernel = FALSE
RF	randomForest 4.7.1.1	Number of trees (ntree) = 500, number of variables sampled at each split (mtry) = sqrt(number of selected features)
SVM	Kernlab 0.9.32	Kernel = radial basis function (RBF), cost = 1, gamma = 1/ncol(data)

Note: GLM = Generalized Linear Model; NB = Naive Bayes; RF = Random Forest; SVM = Support Vector Machine.

SUPPLEMENTARY TABLE S2 The list of all extracted first-order and texture features

Category	Name
First-order feature	firstorderPercentile10
	firstorderPercentile90
	firstorderEnergy
	firstorderEntropy
	firstorderInterquartileRange
	firstorderKurtosis
	firstorderMaximum
	firstorderMeanAbsoluteDeviation
	firstorderMean
	firstorderMedian
	firstorderMinimum
	firstorderRange
	firstorderRobustMeanAbsoluteDeviation
	firstorderRootMeanSquared
	firstorderSkewness
	firstorderTotalEnergy
	firstorderUniformity
	firstorderVariance
Texture feature	glcmAutocorrelation
	glcmClusterProminence
	glcmClusterShade
	glcmClusterTendency
	glcmContrast
	glcmCorrelation
	glcmDifferenceAverage
	glcmDifferenceEntropy
	glcmDifferenceVariance
	glcmId
	glcmIdm
	glcmIdmn
	glcmIdn
	glcmImc1
	glcmImc2
	glcmInverseVariance
	glcmJointAverage
	glcmJointEnergy
	glcmJointEntropy
	glcmMCC
	glcmMaximumProbability
	glcmSumAverage
	glcmSumEntropy
	glcmSumSquares

gldmDependenceEntropy
gldmDependenceNonUniformity
gldmDependenceNonUniformityNormalized
gldmDependenceVariance
gldmGrayLevelNonUniformity
gldmGrayLevelVariance
gldmHighGrayLevelEmphasis
gldmLargeDependenceEmphasis
gldmLargeDependenceHighGrayLevelEmphasis
gldmLargeDependenceLowGrayLevelEmphasis
gldmLowGrayLevelEmphasis
gldmSmallDependenceEmphasis
gldmSmallDependenceHighGrayLevelEmphasis
gldmSmallDependenceLowGrayLevelEmphasis
glrlmGrayLevelNonUniformity
glrlmGrayLevelNonUniformityNormalized
glrlmGrayLevelVariance
glrlmHighGrayLevelRunEmphasis
glrlmLongRunEmphasis
glrlmLongRunHighGrayLevelEmphasis
glrlmLongRunLowGrayLevelEmphasis
glrlmLowGrayLevelRunEmphasis
glrlmRunEntropy
glrlmRunLengthNonUniformity
glrlmRunLengthNonUniformityNormalized
glrlmRunPercentage
glrlmRunVariance
glrlmShortRunEmphasis
glrlmShortRunHighGrayLevelEmphasis
glrlmShortRunLowGrayLevelEmphasis
glszmGrayLevelNonUniformity
glszmGrayLevelNonUniformityNormalized
glszmGrayLevelVariance
glszmHighGrayLevelZoneEmphasis
glszmLargeAreaEmphasis
glszmLargeAreaHighGrayLevelEmphasis
glszmLargeAreaLowGrayLevelEmphasis
glszmLowGrayLevelZoneEmphasis
glszmSizeZoneNonUniformity
glszmSizeZoneNonUniformityNormalized
glszmSmallAreaEmphasis
glszmSmallAreaHighGrayLevelEmphasis
glszmSmallAreaLowGrayLevelEmphasis
glszmZoneEntropy

glszmZonePercentage
glszmZoneVariance
ngtdmBusyness
ngtdmCoarseness
ngtdmComplexity
ngtdmContrast
ngtdmStrength

Note: glcm = gray level co-occurrence matrix; gldm = gray level dependence matrix; glrlm = gray level run length matrix; glszm = gray level size zone matrix; ngtdm = neighborhood gray tone difference matrix.

SUPPLEMENTARY TABLE S3 Comparison of extracted features between DLIR (DLRL, DLRM, DLRH) and AR50/FBP

Comparison group	Feature number ($P \leq 0.05$)	Effect size (Mean |d|)	Feature number (|d| ⩾ 0.5)	Feature number (|d| ⩾ 0.8)	PERMANOVA *P* value
DLRL vs AR50	279	0.314	182	40	0.915
DLRM vs AR50	579	0.634	466	301	0.198
DLRH vs AR50	719	1.323	631	533	0.040
DLRL vs FBP	756	1.863	700	641	0.006
DLRM vs FBP	769	2.427	727	676	0.001
DLRH vs FBP	777	2.940	733	688	0.002

Note: Cohen's d, namely ratio of the mean difference of paired data to the standard deviation of all paired differences, was calculated to measure the practical magnitude of feature differences, with thresholds: $|d| < 0.2$ (trivial), $0.2 \leqslant |d| < 0.5$ (small), $0.5 \leqslant |d| < 0.8$ (medium), $|d| \geqslant 0.8$ (large). The permutational multivariate analysis of variance (PERMANOVA) was performed to test whether the global feature space distribution differed significantly between DLIR and AR50/FBP datasets, and *P* values were adjusted using the False Discovery Rate (FDR) correction. DLIR = deep learning image reconstruction; FBP = filtered back projection; ASIR-V = adaptive statistical iterative reconstruction-Veo; AR50 = ASIR-V at 50% blending level; DLRL/DLRM/DLRH = DLIR at low/medium/high strength.

SUPPLEMENTARY TABLE S4 Comparison of frequently selected 15 features between DLIR (DLRL, DLRM, DLRH) and AR50/FBP

Group	Feature name	|d| (DLRL vs AR50)	|d| (DLRM vs AR50)	|d| (DLRH vs AR50)	|d| (DLRL vs FBP)	|d| (DLRM vs FBP)	|d| (DLRH vs FBP)
DLRL	wavelet_LHL_glszm_ZoneEntropy (16)	0.175 (P = 0.439)	0.342 (P = 0.120)	1.755 (P < 0.001)	0.087 (P = 0.604)	0.462 (P = 0.035)	1.890 (P < 0.001)
	wavelet_LLL_glszm_SizeZoneNonUniformity (13)	0.175 (P = 0.371)	0.119 (P = 0.468)	0.408 (P = 0.010)	0.881 (P < 0.001)	1.148 (P < 0.001)	1.405 (P < 0.001)
	wavelet_HHL_firstorder_Skewness (12)	0.504 (P = 0.008)	0.682 (P < 0.001)	0.862 (P < 0.001)	0.364 (P = 0.026)	0.573 (P = 0.003)	0.791 (P < 0.001)
	wavelet_LHL_firstorder_Mean (11)	0.084 (P = 0.707)	0.073 (P = 0.682)	0.062 (P = 0.716)	0.046 (P = 0.780)	0.035 (P = 0.815)	0.024 (P = 0.880)
	original_glszm_ZonePercentage (10)	0.476 (P = 0.009)	0.366 (P = 0.028)	1.328 (P < 0.001)	2.577 (P < 0.001)	3.393 (P < 0.001)	4.274 (P < 0.001)
	wavelet_LHL_glszm_LargeAreaHighGrayLevelEmphasis (10)	0.412 (P = 0.017)	0.012 (P = 0.793)	0.591 (P < 0.001)	0.962 (P < 0.001)	1.134 (P < 0.001)	1.330 (P < 0.001)
	wavelet_LLH_glcm_Imc1 (9)	0.114 (P = 0.309)	0.256 (P = 0.343)	0.697 (P < 0.001)	1.088 (P < 0.001)	1.374 (P < 0.001)	1.781 (P < 0.001)
	wavelet_HHH_firstorder_Skewness (8)	0.282 (P = 0.121)	0.227 (P = 0.089)	0.094 (P = 0.061)	0.101 (P = 0.313)	0.057 (P = 0.389)	0.024 (P = 0.318)
	wavelet_LLH_glszm_GrayLevelNonUniformity (7)	0.141 (P = 0.707)	0.878 (P < 0.001)	1.835 (P < 0.001)	1.314 (P < 0.001)	2.603 (P < 0.001)	3.615 (P < 0.001)
	wavelet_HHH_firstorder_Minimum (6)	0.167 (P = 0.376)	1.040 (P < 0.001)	1.922 (P < 0.001)	2.929 (P < 0.001)	3.679 (P < 0.001)	4.383 (P < 0.001)
	wavelet_HLL_gldm_SmallDependenceLowGrayLevelEmphasis (6)	0.168 (P = 0.179)	0.054 (P = 0.535)	0.420 (P = 0.003)	0.119 (P = 0.205)	0.252 (P = 0.025)	0.794 (P < 0.001)
	wavelet_LLL_gldm_SmallDependenceLowGrayLevelEmphasis (6)	0.125 (P = 0.776)	0.218 (P = 0.153)	0.342 (P = 0.017)	0.574 (P < 0.001)	0.652 (P < 0.001)	0.767 (P < 0.001)
	original_ngtdm_Contrast (5)	0.217 (P = 0.210)	0.389 (P = 0.007)	0.968 (P < 0.001)	1.776 (P < 0.001)	2.405 (P < 0.001)	2.883 (P < 0.001)
	wavelet_HLH_firstorder_Maximum (4)	0.528 (P = 0.003)	1.264 (P < 0.001)	1.927 (P < 0.001)	2.937 (P < 0.001)	3.574 (P < 0.001)	4.134 (P < 0.001)
	wavelet_LHL_glrlm_RunEntropy (4)	0.169 (P = 0.458)	0.239 (P = 0.124)	0.322 (P = 0.049)	2.505 (P < 0.001)	2.704 (P < 0.001)	2.383 (P < 0.001)
DLRM	wavelet_LLL_gldm_SmallDependenceLowGrayLevelEmphasis (22)	0.125 (P = 0.776)	0.218 (P = 0.153)	0.342 (P = 0.017)	0.574 (P < 0.001)	0.652 (P < 0.001)	0.767 (P < 0.001)
	wavelet_LHH_glszm_SmallAreaEmphasis (15)	0.301 (P = 0.143)	0.482 (P = 0.005)	1.323 (P < 0.001)	3.612 (P < 0.001)	3.705 (P < 0.001)	0.933 (P < 0.001)
	wavelet_LLL_glszm_SizeZoneNonUniformity (15)	0.175 (P = 0.371)	0.119 (P = 0.468)	0.408 (P = 0.010)	0.881 (P < 0.001)	1.148 (P < 0.001)	1.405 (P < 0.001)
	wavelet_LHH_glszm_SizeZoneNonUniformityNormalized (12)	0.322 (P = 0.183)	0.491 (P = 0.004)	1.386 (P < 0.001)	3.408 (P < 0.001)	3.441 (P < 0.001)	1.145 (P < 0.001)
	wavelet_LHL_glszm_LargeAreaHighGrayLevelEmphasis (11)	0.412 (P = 0.017)	0.012 (P = 0.793)	0.591 (P < 0.001)	0.962 (P < 0.001)	1.134 (P < 0.001)	1.330 (P < 0.001)
	wavelet_LLH_glszm_ZoneEntropy (7)	0.421 (P = 0.030)	0.213 (P = 0.239)	0.398 (P = 0.017)	1.082 (P < 0.001)	0.833 (P < 0.001)	0.207 (P = 0.206)
	wavelet_LHL_glrlm_RunEntropy (7)	0.169 (P = 0.458)	0.239 (P = 0.124)	0.322 (P = 0.049)	2.505 (P < 0.001)	2.704 (P < 0.001)	2.383 (P < 0.001)
	wavelet_HHH_firstorder_Minimum (6)	0.167 (P = 0.376)	1.040 (P < 0.001)	1.922 (P < 0.001)	2.929 (P < 0.001)	3.679 (P < 0.001)	4.383 (P < 0.001)
	wavelet_HHL_firstorder_Median (6)	0.226 (P = 0.287)	0.295 (P = 0.089)	0.330 (P = 0.046)	0.109 (P = 0.513)	0.143 (P = 0.386)	0.160 (P = 0.329)
	wavelet_LHL_glszm_ZoneEntropy (5)	0.175 (P = 0.439)	0.342 (P = 0.120)	1.755 (P < 0.001)	0.087 (P = 0.604)	0.462 (P = 0.035)	1.890 (P < 0.001)
	wavelet_LHL_firstorder_Mean (5)	0.084 (P = 0.707)	0.073 (P = 0.682)	0.062 (P = 0.716)	0.046 (P = 0.780)	0.035 (P = 0.815)	0.024 (P = 0.880)
	wavelet_HHL_firstorder_Skewness (4)	0.504 (P = 0.008)	0.682 (P < 0.001)	0.862 (P < 0.001)	0.364 (P = 0.026)	0.573 (P = 0.003)	0.791 (P < 0.001)
	wavelet_HHH_ngtdm_Busyness (4)	0.235 (P = 0.235)	1.528 (P < 0.001)	2.469 (P < 0.001)	0.831 (P < 0.001)	2.143 (P < 0.001)	3.572 (P < 0.001)
	wavelet_HHH_glszm_GrayLevelNonUniformity (4)	0.127 (P = 0.464)	0.694 (P < 0.001)	0.647 (P = 0.001)	2.059 (P < 0.001)	2.074 (P < 0.001)	2.073 (P < 0.001)
	original_glszm_ZonePercentage (4)	0.476 (P = 0.009)	0.366 (P = 0.028)	1.328 (P < 0.001)	2.577 (P < 0.001)	3.393 (P < 0.001)	4.274 (P < 0.001)

DLRH	wavelet_HHL_glszm_SmallAreaEmphasis (24)	0.624 (P < 0.001)	0.850 (P < 0.001)	1.653 (P < 0.001)	0.347 (P = 0.005)	1.417 (P < 0.001)	2.454 (P < 0.001)
	wavelet_HHH_firstorder_Maximum (19)	0.390 (P = 0.047)	1.977 (P < 0.001)	3.652 (P < 0.001)	3.921 (P < 0.001)	5.060 (P < 0.001)	6.131 (P < 0.001)
	wavelet_LLL_glszm_SizeZoneNonUniformity (13)	0.175 (P = 0.371)	0.119 (P = 0.468)	0.408 (P = 0.010)	0.881 (P < 0.001)	1.148 (P < 0.001)	1.405 (P < 0.001)
	wavelet_HLL_glszm_ZoneEntropy (7)	0.521 (P = 0.006)	0.621 (P < 0.001)	1.032 (P < 0.001)	0.799 (P < 0.001)	2.092 (P < 0.001)	2.542 (P < 0.001)
	wavelet_LLL_gldm_SmallDependenceLowGrayLevelEmphasis (7)	0.125 (P = 0.776)	0.218 (P = 0.153)	0.342 (P = 0.017)	0.574 (P < 0.001)	0.652 (P < 0.001)	0.767 (P < 0.001)
	wavelet_LLL_glcm_InverseVariance (6)	0.068 (P = 0.665)	0.196 (P = 0.281)	0.986 (P < 0.001)	1.958 (P < 0.001)	2.145 (P < 0.001)	1.193 (P < 0.001)
	wavelet_LHH_firstorder_Uniformity (6)	0.177 (P = 0.549)	1.527 (P < 0.001)	2.062 (P < 0.001)	3.639 (P < 0.001)	4.258 (P < 0.001)	4.392 (P < 0.001)
	wavelet_LLL_glcm_Imc1 (6)	0.649 (P < 0.001)	0.654 (P < 0.001)	2.395 (P < 0.001)	2.336 (P < 0.001)	3.595 (P < 0.001)	5.219 (P < 0.001)
	wavelet_LHH_glszm_ZonePercentage (5)	0.283 (P = 0.271)	1.608 (P < 0.001)	2.310 (P < 0.001)	4.779 (P < 0.001)	6.881 (P < 0.001)	7.359 (P < 0.001)
	wavelet_LHL_glszm_GrayLevelNonUniformity (5)	0.056 (P = 0.831)	0.238 (P = 0.177)	0.299 (P = 0.038)	0.900 (P < 0.001)	0.583 (P < 0.001)	1.078 (P < 0.001)
	wavelet_LHL_glcm_Correlation (5)	0.541 (P = 0.002)	0.036 (P = 0.825)	0.669 (P < 0.001)	1.031 (P < 0.001)	1.466 (P < 0.001)	2.072 (P < 0.001)
	wavelet_LHL_glszm_LargeAreaHighGrayLevelEmphasis (5)	0.412 (P = 0.017)	0.012 (P = 0.793)	0.591 (P < 0.001)	0.962 (P < 0.001)	1.134 (P < 0.001)	1.330 (P < 0.001)
	original_glszm_ZonePercentage (4)	0.476 (P = 0.009)	0.366 (P = 0.028)	1.328 (P < 0.001)	2.577 (P < 0.001)	3.393 (P < 0.001)	4.274 (P < 0.001)
	wavelet_HHL_glcm_ClusterShade (4)	0.471 (P = 0.014)	0.612 (P < 0.001)	0.786 (P < 0.001)	0.124 (P = 0.457)	0.142 (P = 0.389)	0.185 (P = 0.019)
	wavelet_LLH_glszm_SmallAreaEmphasis (4)	0.427 (P = 0.027)	0.524 (P = 0.002)	0.352 (P = 0.017)	1.226 (P < 0.001)	1.301 (P < 0.001)	0.301 (P = 0.301)
AR50	wavelet_LHL_firstorder_Maximum (12)	0.201 (P = 0.340)	0.249 (P = 0.168)	0.677 (P < 0.001)	1.496 (P < 0.001)	1.952 (P < 0.001)	2.397 (P < 0.001)
	wavelet_LHH_glszm_ZoneEntropy (11)	0.611 (P = 0.002)	0.442 (P = 0.026)	1.676 (P < 0.001)	3.388 (P < 0.001)	2.205 (P < 0.001)	1.215 (P < 0.001)
	wavelet_LLH_glcm_Imc1 (11)	0.114 (P = 0.309)	0.256 (P = 0.343)	0.697 (P < 0.001)	1.088 (P < 0.001)	1.374 (P < 0.001)	1.781 (P < 0.001)
	wavelet_LLL_glszm_SizeZoneNonUniformity (10)	0.175 (P = 0.371)	0.119 (P = 0.468)	0.408 (P = 0.010)	0.881 (P < 0.001)	1.148 (P < 0.001)	1.405 (P < 0.001)
	wavelet_HHL_firstorder_Skewness (9)	0.504 (P = 0.008)	0.682 (P < 0.001)	0.862 (P < 0.001)	0.364 (P = 0.026)	0.573 (P = 0.003)	0.791 (P < 0.001)
	wavelet_LHH_firstorder_Kurtosis (9)	1.651 (P < 0.001)	1.034 (P < 0.001)	0.089 (P < 0.001)	0.993 (P < 0.001)	0.291 (P < 0.001)	0.355 (P = 0.959)
	wavelet_LHH_firstorder_Median (9)	0.524 (P = 0.015)	0.529 (P = 0.002)	0.344 (P = 0.038)	0.160 (P = 0.466)	0.136 (P = 0.407)	0.018 (P = 0.917)
	original_glszm_ZonePercentage (8)	0.476 (P = 0.009)	0.366 (P = 0.028)	1.328 (P < 0.001)	2.577 (P < 0.001)	3.393 (P < 0.001)	4.274 (P < 0.001)
	wavelet_LHL_glrlm_RunEntropy (8)	0.169 (P = 0.458)	0.239 (P = 0.124)	0.322 (P = 0.049)	2.505 (P < 0.001)	2.704 (P < 0.001)	2.383 (P < 0.001)
	wavelet_LLH_glszm_GrayLevelNonUniformity (8)	0.141 (P = 0.707)	0.878 (P < 0.001)	1.835 (P < 0.001)	1.314 (P < 0.001)	2.603 (P < 0.001)	3.615 (P < 0.001)
	wavelet_HLH_glszm_LargeAreaHighGrayLevelEmphasis (7)	0.188 (P = 0.458)	1.083 (P < 0.001)	1.124 (P < 0.001)	1.334 (P < 0.001)	1.770 (P < 0.001)	2.076 (P < 0.001)
	wavelet_HHH_gldm_SmallDependenceHighGrayLevelEmphasis (7)	0.292 (P = 0.179)	1.487 (P < 0.001)	2.151 (P < 0.001)	1.990 (P < 0.001)	2.151 (P < 0.001)	2.206 (P < 0.001)
	wavelet_HHH_firstorder_Skewness (6)	0.282 (P = 0.121)	0.227 (P = 0.089)	0.094 (P = 0.061)	0.101 (P = 0.313)	0.057 (P = 0.389)	0.024 (P = 0.318)
	wavelet_LLL_gldm_SmallDependenceLowGrayLevelEmphasis (6)	0.125 (P = 0.776)	0.218 (P = 0.153)	0.342 (P = 0.017)	0.574 (P < 0.001)	0.652 (P < 0.001)	0.767 (P < 0.001)
	wavelet_HHH_firstorder_Minimum (5)	0.167 (P = 0.376)	1.040 (P < 0.001)	1.922 (P < 0.001)	2.929 (P < 0.001)	3.679 (P < 0.001)	4.383 (P < 0.001)
FBP	wavelet_LLH_gldm_DependenceVariance (28)	0.540 (P = 0.004)	0.619 (P < 0.001)	0.266 (P = 0.040)	0.409 (P = 0.013)	0.509 (P < 0.001)	0.090 (P = 0.142)
	wavelet_LLL_glszm_SmallAreaEmphasis (22)	0.171 (P = 0.404)	0.153 (P = 0.314)	0.144 (P = 0.306)	0.078 (P = 0.839)	0.087 (P = 0.857)	0.086 (P = 0.793)

	wavelet_HHL_glcm_ClusterShade (13)	0.471 (P = 0.014)	0.612 (P < 0.001)	0.786 (P < 0.001)	0.124 (P = 0.457)	0.142 (P = 0.389)	0.185 (P = 0.019)
	wavelet_LHH_ngtdm_Strength (13)	0.419 (P = 0.031)	0.773 (P < 0.001)	0.979 (P < 0.001)	1.913 (P < 0.001)	2.176 (P < 0.001)	2.334 (P < 0.001)
	wavelet_HHH_firstorder_Skewness (9)	0.282 (P = 0.121)	0.227 (P = 0.089)	0.094 (P = 0.061)	0.101 (P = 0.313)	0.057 (P = 0.389)	0.024 (P = 0.318)
	wavelet_LHL_firstorder_Mean (8)	0.084 (P = 0.707)	0.073 (P = 0.682)	0.062 (P = 0.716)	0.046 (P = 0.780)	0.035 (P = 0.815)	0.024 (P = 0.880)
	wavelet_LLH_glrlm_RunEntropy (7)	0.338 (P = 0.093)	0.443 (P = 0.010)	0.361 (P = 0.029)	0.432 (P = 0.009)	0.542 (P < 0.001)	0.440 (P = 0.007)
	wavelet_LHL_firstorder_Maximum (7)	0.201 (P = 0.340)	0.249 (P = 0.168)	0.677 (P < 0.001)	1.496 (P < 0.001)	1.952 (P < 0.001)	2.397 (P < 0.001)
	wavelet_LHL_glszm_LargeAreaHighGrayLevelEmphasis (6)	0.412 (P = 0.017)	0.012 (P = 0.793)	0.591 (P < 0.001)	0.962 (P < 0.001)	1.134 (P < 0.001)	1.330 (P < 0.001)
	wavelet_LLH_gldm_DependenceNonUniformityNormalized (6)	0.583 (P = 0.002)	0.545 (P < 0.001)	0.062 (P = 0.739)	0.840 (P < 0.001)	0.779 (P < 0.001)	0.214 (P = 0.191)
	wavelet_HLH_glcm_ClusterShade (6)	0.670 (P < 0.001)	0.899 (P < 0.001)	0.976 (P < 0.001)	0.621 (P < 0.001)	0.762 (P < 0.001)	0.812 (P < 0.001)
	wavelet_HHL_firstorder_Skewness (5)	0.504 (P = 0.008)	0.682 (P < 0.001)	0.862 (P < 0.001)	0.364 (P = 0.026)	0.573 (P = 0.003)	0.791 (P < 0.001)
	wavelet_HLH_firstorder_Maximum (4)	0.528 (P = 0.003)	1.264 (P < 0.001)	1.927 (P < 0.001)	2.937 (P < 0.001)	3.574 (P < 0.001)	4.134 (P < 0.001)
	wavelet_LHL_glszm_SizeZoneNonUniformityNormalized (4)	0.706 (P < 0.001)	1.157 (P < 0.001)	0.071 (P = 0.698)	3.571 (P < 0.001)	7.246 (P < 0.001)	4.348 (P < 0.001)
	wavelet_HLH_firstorder_Mean (4)	0.050 (P = 0.721)	0.021 (P = 0.842)	0.015 (P = 0.966)	0.015 (P = 0.975)	0.043 (P = 0.751)	0.079 (P = 0.567)

Note: Cohen’s d, namely ratio of the "mean difference" of paired data to the standard deviation of all paired differences, was calculated to measure the practical magnitude of feature differences, with thresholds: |d| < 0.2 (trivial), 0.2 ≤ |d| < 0.5 (small), 0.5 ≤ |d| < 0.8 (medium), |d| ≥ 0.8 (large). *P* value indicates the results of comparisons between DLIR (DLRL, DLRM, DLRH) and AR50/FBP using independent t-test or Wilcoxon rank-sum test, and *P* values were adjusted using the False Discovery Rate (FDR) correction.

SUPPLEMENTARY TABLE S5 Model performance transfer drop from DLIR datasets to AR50 and FBP datasets between different ML-based radiomics models

Source dataset	Target dataset	Model1	Model2	Model AUC Drop1	Model AUC Drop2	Drop1-Drop2	*P* value
DLRL	AR50	GLM	NB	-0.132 (-0.158 – -0.106)	-0.101 (-0.130 – -0.072)	-0.031	0.273
DLRL	AR50	GLM	RF	-0.132 (-0.158 – -0.106)	-0.102 (-0.125 – -0.079)	-0.030	0.250
DLRL	AR50	GLM	SVM	-0.132 (-0.158 – -0.106)	-0.126 (-0.150 – -0.103)	-0.006	0.856
DLRL	AR50	NB	RF	-0.101 (-0.130 – -0.072)	-0.102 (-0.125 – -0.079)	0.001	0.944
DLRL	AR50	NB	SVM	-0.101 (-0.130 – -0.072)	-0.126 (-0.150 – -0.103)	0.025	0.358
DLRL	AR50	RF	SVM	-0.102 (-0.125 – -0.079)	-0.126 (-0.150 – -0.103)	0.024	0.316
DLRL	FBP	GLM	NB	-0.037 (-0.065 – -0.008)	0.014 (-0.030 – 0.057)	-0.051	0.197
DLRL	FBP	GLM	RF	-0.037 (-0.065 – -0.008)	0.024 (-0.006 – 0.053)	-0.061	0.067
DLRL	FBP	GLM	SVM	-0.037 (-0.065 – -0.008)	-0.030 (-0.061 – 0.001)	-0.007	0.856
DLRL	FBP	NB	RF	0.014 (-0.030 – 0.057)	0.024 (-0.006 – 0.053)	-0.010	0.856
DLRL	FBP	NB	SVM	0.014 (-0.030 – 0.057)	-0.030 (-0.061 – 0.001)	0.044	0.273
DLRL	FBP	RF	SVM	0.024 (-0.006 – 0.053)	-0.030 (-0.061 – 0.001)	0.054	0.084
DLRM	AR50	GLM	NB	-0.039 (-0.080 – 0.003)	0.057 (-0.006 – 0.119)	-0.096	0.084
DLRM	AR50	GLM	RF	-0.039 (-0.080 – 0.003)	-0.033 (-0.067 – 0.002)	-0.006	0.900
DLRM	AR50	GLM	SVM	-0.039 (-0.080 – 0.003)	-0.046 (-0.086 – -0.006)	0.007	0.889
DLRM	AR50	NB	RF	0.057 (-0.006 – 0.119)	-0.033 (-0.067 – 0.002)	0.090	0.084
DLRM	AR50	NB	SVM	0.057 (-0.006 – 0.119)	-0.046 (-0.086 – -0.006)	0.103	0.079
DLRM	AR50	RF	SVM	-0.033 (-0.067 – 0.002)	-0.046 (-0.086 – -0.006)	0.013	0.784
DLRM	FBP	GLM	NB	0.144 (0.087 – 0.200)	0.120 (0.081 – 0.159)	0.024	0.771
DLRM	FBP	GLM	RF	0.144 (0.087 – 0.200)	0.165 (0.107 – 0.223)	-0.021	0.784
DLRM	FBP	GLM	SVM	0.144 (0.087 – 0.200)	0.138 (0.079 – 0.196)	0.006	0.938
DLRM	FBP	NB	RF	0.120 (0.081 – 0.159)	0.165 (0.107 – 0.223)	-0.045	0.388
DLRM	FBP	NB	SVM	0.120 (0.081 – 0.159)	0.138 (0.079 – 0.196)	-0.018	0.784
DLRM	FBP	RF	SVM	0.165 (0.107 – 0.223)	0.138 (0.079 – 0.196)	0.027	0.771
DLRH	AR50	GLM	NB	0.085 (0.039 – 0.131)	0.147 (0.093 – 0.200)	-0.062	0.250
DLRH	AR50	GLM	RF	0.085 (0.039 – 0.131)	0.041 (0.004 – 0.079)	0.044	0.316
DLRH	AR50	GLM	SVM	0.085 (0.039 – 0.131)	0.062 (0.009 – 0.115)	0.023	0.771
DLRH	AR50	NB	RF	0.147 (0.093 – 0.200)	0.041 (0.004 – 0.079)	0.106	0.067
DLRH	AR50	NB	SVM	0.147 (0.093 – 0.200)	0.062 (0.009 – 0.115)	0.085	0.132
DLRH	AR50	RF	SVM	0.041 (0.004 – 0.079)	0.062 (0.009 – 0.115)	-0.021	0.771
DLRH	FBP	GLM	NB	0.224 (0.180 – 0.267)	0.164 (0.125 – 0.203)	0.060	0.191
DLRH	FBP	GLM	RF	0.224 (0.180 – 0.267)	0.166 (0.125 – 0.207)	0.058	0.197
DLRH	FBP	GLM	SVM	0.224 (0.180 – 0.267)	0.204 (0.151 – 0.258)	0.020	0.784
DLRH	FBP	NB	RF	0.164 (0.125 – 0.203)	0.166 (0.125 – 0.207)	-0.002	0.944
DLRH	FBP	NB	SVM	0.164 (0.125 – 0.203)	0.204 (0.151 – 0.258)	-0.040	0.414
DLRH	FBP	RF	SVM	0.166 (0.125 – 0.207)	0.204 (0.151 – 0.258)	-0.038	0.450

Note: *P* value indicates the results of comparisons using independent t-test for AUC drop among different machine learning models of each paired transfer from DLIR (DLRL, DLRM, DLRH) to AR50 and FBP datasets, and *P* values were adjusted using the False Discovery Rate (FDR) correction.

SUPPLEMENTARY TABLE S6 Model performance transfer drop from AR50 and FBP datasets to DLIR datasets between different ML-based radiomics models

Source dataset	Target dataset	Model1	Model2	Model AUC Drop1	Model AUC Drop2	Drop1-Drop2	*P* value
AR50	DLRL	GLM	NB	-0.109(-0.140 – -0.077)	-0.083 (-0.109 – -0.056)	-0.026	0.377
AR50	DLRL	GLM	RF	-0.109(-0.140 – -0.077)	-0.115 (-0.138 – -0.093)	0.006	0.870
AR50	DLRL	GLM	SVM	-0.109(-0.140 – -0.077)	-0.112 (-0.142 – -0.082)	0.003	0.932
AR50	DLRL	NB	RF	-0.083 (-0.109 – -0.056)	-0.115 (-0.138 – -0.093)	0.032	0.139
AR50	DLRL	NB	SVM	-0.083 (-0.109 – -0.056)	-0.112 (-0.142 – -0.082)	0.029	0.296
AR50	DLRL	RF	SVM	-0.115 (-0.138 – -0.093)	-0.112 (-0.142 – -0.082)	-0.003	0.928
FBP	DLRL	GLM	NB	-0.104(-0.143 – -0.065)	-0.049 (-0.090 – -0.008)	-0.055	0.135
FBP	DLRL	GLM	RF	-0.104(-0.143 – -0.065)	-0.123 (-0.151 – -0.096)	0.019	0.609
FBP	DLRL	GLM	SVM	-0.104(-0.143 – -0.065)	-0.093 (-0.126 – -0.060)	-0.011	0.870
FBP	DLRL	NB	RF	-0.049 (-0.090 – -0.008)	-0.123 (-0.151 – -0.096)	0.074	0.010
FBP	DLRL	NB	SVM	-0.049 (-0.090 – -0.008)	-0.093 (-0.126 – -0.060)	0.044	0.207
FBP	DLRL	RF	SVM	-0.123 (-0.151 – -0.096)	-0.093 (-0.126 – -0.060)	-0.030	0.312
AR50	DLRM	GLM	NB	-0.105(-0.141 – -0.069)	-0.026 (-0.067 – 0.015)	-0.079	0.013
AR50	DLRM	GLM	RF	-0.105(-0.141 – -0.069)	-0.097 (-0.120 – -0.075)	-0.008	0.870
AR50	DLRM	GLM	SVM	-0.105(-0.141 – -0.069)	-0.111 (-0.144 – -0.078)	0.006	0.878
AR50	DLRM	NB	RF	-0.026 (-0.067 – 0.015)	-0.097 (-0.120 – -0.075)	0.071	0.010
AR50	DLRM	NB	SVM	-0.026 (-0.067 – 0.015)	-0.111 (-0.144 – -0.078)	0.085	0.007
AR50	DLRM	RF	SVM	-0.097 (-0.120 – -0.075)	-0.111 (-0.144 – -0.078)	0.014	0.654
FBP	DLRM	GLM	NB	-0.107(-0.146 – -0.067)	-0.013 (-0.054 – 0.028)	-0.094	0.007
FBP	DLRM	GLM	RF	-0.107(-0.146 – -0.067)	-0.116 (-0.143 – -0.089)	0.009	0.870
FBP	DLRM	GLM	SVM	-0.107(-0.146 – -0.067)	-0.099 (-0.133 – -0.065)	-0.008	0.878
FBP	DLRM	NB	RF	-0.013 (-0.054 – 0.028)	-0.116 (-0.143 – -0.089)	0.103	< 0.001
FBP	DLRM	NB	SVM	-0.013 (-0.054 – 0.028)	-0.099 (-0.133 – -0.065)	0.086	0.007
FBP	DLRM	RF	SVM	-0.116 (-0.143 – -0.089)	-0.099 (-0.133 – -0.065)	-0.017	0.609
AR50	DLRH	GLM	NB	-0.089(-0.126 – -0.052)	0.038 (-0.019 – 0.095)	-0.127	0.003
AR50	DLRH	GLM	RF	-0.089(-0.126 – -0.052)	-0.061 (-0.089 – -0.034)	-0.028	0.409
AR50	DLRH	GLM	SVM	-0.089(-0.126 – -0.052)	-0.088 (-0.131 – -0.046)	-0.001	0.980
AR50	DLRH	NB	RF	0.038 (-0.019 – 0.095)	-0.061 (-0.089 – -0.034)	0.099	0.009
AR50	DLRH	NB	SVM	0.038 (-0.019 – 0.095)	-0.088 (-0.131 – -0.046)	0.126	0.004
AR50	DLRH	RF	SVM	-0.061 (-0.089 – -0.034)	-0.088 (-0.131 – -0.046)	0.027	0.484
FBP	DLRH	GLM	NB	-0.082(-0.129 – -0.036)	0.064 (0.017 – 0.110)	-0.146	< 0.001
FBP	DLRH	GLM	RF	-0.082(-0.129 – -0.036)	-0.104 (-0.133 – -0.076)	0.022	0.609
FBP	DLRH	GLM	SVM	-0.082(-0.129 – -0.036)	-0.080 (-0.122 – -0.037)	-0.002	0.953
FBP	DLRH	NB	RF	0.064 (0.017 – 0.110)	-0.104 (-0.133 – -0.076)	0.168	< 0.001
FBP	DLRH	NB	SVM	0.064 (0.017 – 0.110)	-0.080 (-0.122 – -0.037)	0.144	< 0.001
FBP	DLRH	RF	SVM	-0.104 (-0.133 – -0.076)	-0.080 (-0.122 – -0.037)	-0.024	0.532

Note: *P* value indicates the results of comparisons using independent t-test for AUC drop among different machine learning models of each paired transfer from AR50 and FBP to DLIR (DLRL, DLRM, DLRH) datasets, and *P* values were adjusted using the False Discovery Rate (FDR) correction.
